# Otoacoustic emissions value in patients with idiopathic sudden sensorineural hearing loss

**DOI:** 10.1016/j.joto.2022.06.001

**Published:** 2022-06-23

**Authors:** Aya El-sayed El-sayed Gaafar, Elshahat Ibrahem Ismail, Hesham Saad Zaghloul

**Affiliations:** a Ministry of Health, Egypt; b ENT Department, Faculty of Medicine, Mansoura University, Egypt

**Keywords:** Sudden sensorineural hearing loss, Transiently evoked otoacoustic emission, Distortion product otoacoustic emission, Outer hair cells

## Abstract

**Objectives:**

This study aimed to determine the prognostic value of **otoacoustic emissions** (OAEs) in idiopathic sudden sensorineural hearing loss patients.

**Methods:**

The study included 30 subjects with unilateral idiopathic sudden sensorineural hearing loss (ISSNHL). Each patient was evaluated four times: at baseline and after one week, one month, and three months of treatment. During each visit, each patient was subjected to full audiological history, otoscopic examination, basic audiological evaluations, and transiently evoked and distortion product otoacoustic emission (TEOAEs & DEOAEs).

**Results:**

The hearing thresholds (frequency range 250–8000 Hz) and word recognition scores of patients with detectable TEOAEs and DPOAEs improved significantly, whereas no significant improvements were observed in those with no response.

**Conclusion:**

Hearing improvement is better in patients with detectable TEOAEs and DPOAEs. As a result, TEOAEs and DPOAEs are recommended as routine tests in all SSNHL patients to predict outcomes and monitor treatment as TEOAEs and DPOAEs reflect the cochlear OHCs activity.

## 1 Introduction

Sudden sensorineural hearing loss (SSNHL) is defined as a 30 dB or more sensorineural hearing loss over at least three consecutive audiometric frequencies within 72 h **(****Kuhn et al., 2011****)**. It is a common condition, affecting 1.5–1.7 per 100 new patients **(****Chau et al., 2010**; **Huy et al., 2005**; **Nosrati-Zarenoe et al., 2007****).**

Most SSNHL patients are idiopathic **(****Chau et al., 2010**; **Nosrati-Zarenoe et al., 2007****)**. The cause and proper treatment are still unknown. Hearing threshold recovery may be total or partial regardless of the cause. Factors affecting recovery are the patient's age at hearing loss onset, severity and frequency ranges, vertigo, and the interval between hearing loss onset and the first contact with the treating physician **(****Kuhn et al., 2011****).**

The underlying etiology is known in 7%–45% of SSNHL patients **(****Chau et al., 2010**; **Huy et al., 2005**; **Nosrati-Zarenoe et al., 2007****)**. The differential diagnosis is extensive; infectious causes account for 13%, followed by otologic (5%), traumatic (4%), vascular or hematologic (3%), neoplastic (2%), and miscellaneous (2%) causes **(****Chau et al., 2010****)**. Hearing loss occurs due to damage to hair cells or other cochlear structures in many of these etiologies and is permanent. More harm can be avoided if the underlying cause is determined and treated immediately **(****Kuhn et al., 2011****).**

SSNHL treatment should be prompt and focused on the conditions most likely to benefit. After 30 days, treatment may be ineffective since the acute disease may have subsided, and long-term harm may have occurred **(****Vijayendra et al., 2012****)**. Due to its potent anti-inflammatory action, systemic steroid therapy at high doses is the foundation of treatment **(****Choung et al., 2009****)**. Additionally, steroids inhibit cytotoxic immune responses, enhance microvascular blood flow in the cochlea, and delay endolymphatic hydrops development. Antiviral medicines such as acyclovir or valacyclovir should be administered if a viral cause is suspected **(****Vijayendra et al., 2012****).**

Sounds originating in the cochlea can be detected and recorded using a microphone placed in the external auditory canal. Numerous studies evaluated the use of these emissions, termed otoacoustic emissions (OAEs). Transient evoked otoacoustic emissions (TEOAEs) and distortion product otoacoustic emissions (DPOAEs) are detectable in nearly all people with normal cochlear and middle ear function. In contrast, TEOAEs are absent in mild sensorineural hearing loss, and DPOAEs are absent in sensorineural hearing loss greater than 50 dB but detectable in inflammatory diseases associated with cochlear nerve involvement **(****Ishida et al., 2008**; **Lonsbury-Martin et al., 2003****)**. Elicited OAEs may successfully distinguish between normal and hearing-impaired populations **(****Canale et al., 2005**; **Chen, 2006**; **Vaden et al., 2018**; **Gunes et al., 2019**; **Jedrzejczak et al., 2022****).**

Since TEOAEs and DPOAEs indicate outer hair cell (OHC) activity, it can be hypothesized that in the majority of ISSNHL patients, OHC function worsens as the hearing threshold increases and recovers when hearing improves **(****Nemati et al., 2011****).** However, changes in OAEs frequently precede changes in audiometric hearing thresholds. Otoacoustic emissions (OAEs) are derived from the electrochemical motility of the OHC. They indicate the integrity of the inner ear, particularly the cochlear amplifier, and thus they may be beneficial in evaluating the ISSNHL prognosis **(****Babich and Dunckley, 2020****).**

According to Babich and Dunckly, 12 of 14 studies support using OAEs as a diagnostic method for predicting hearing improvement in ISSNHL patients. Some studies used TEOAEs or DPOAEs only, while others used both TEOAEs and DPOAEs.

Shupak et al. (2014) stated that ISSNHL patients with present TEOAEs and DPOAEs in the acute stages demonstrated significant hearing improvement. Moreover, for both TEOAEs and DPOAEs, the specificity and sensitivity of one weak follow-up in hearing prediction improvement were statistically significant, indicating their effectiveness in outcome prediction. Most studies used only one type of OAEs (TEOAEs or DPOAEs) to predict ISSNHL outcomes. Consequently, in this study, we used both TEOAEs and DPOAEs to evaluate the prognostic value of both emissions in ISSNHL patients and the advantages of one type of OAEs over the other.

This study aimed to evaluate the predictive value of TEOAEs and DPOAEs in ISSNHL patients and monitor their changes during recovery.

## 2 Material and methods

This prospective study was conducted in the interval between March 2018 to January 2020 in the Audiology Unit, Mansoura University Hospital. Written informed consent was obtained from all patients following the Helsinki Declaration. The study was conducted following the standards of the Mansoura ORL Department Ethical Committee and was approved by the Faculty of Medicine Institutional Research Board.

Thirty adult patients aged 20–50 years with idiopathic acute sensorineural hearing loss were included within the first week of the attack. All patients showed SNHL of at least 30 dB in three consecutive frequencies within three days.

Patients with a medical or neurological condition affecting the auditory system, a family history of hearing impairment, otoscopic evidence of ear-drum abnormalities, ototoxic drug intake, noise exposure, vertigo, chronic middle ear pathology, or previous ear surgery were excluded.

## 3 Equipment

1 Sound-treated room - locally made.
2 Pure tone audiometer: Madsen-Itera II (Denmark).
3 Impedance audiometer (Interacoustics-AT235, Denmark).
4 Otoacoustic emissions (Biologic Scout OAE, Natus hearing diagnostic version 4.0 USA).

All patients were evaluated four times, during the first week and after one week, one month, and three months of treatment.

Once diagnosed, all patients received oral prednisone (1 mg/kg/day) for seven days, followed by another seven days to taper down the dosage. Intratympanic dexamethasone (10 mg/ml) was injected in the affected ear daily until no significant improvement was observed by pure tone audiometry (after every three intratympanic steroid injections). All patients were referred for a magnetic resonance imaging study of brain and cerebellopontine angle to rule out retrocochlear or brain lesions such as vestibular schwannoma.

During each visit, each patient was subjected to full audiological history, otoscopic examination, and basic audiological evaluations such as pure-tone audiometry (PTA), speech audiometry, and immittancemetry. PTA included air conduction threshold ranging from 250 to 8000 Hz and bone conduction threshold ranging from 500 to 4000 Hz. Speech audiometry included speech recognition threshold (SRT) with Arabic spondee words **(****Soliman et al., 1985****)** and word recognition score with Arabic phonetically balanced words **(****Soliman, 1976****)**. Immittancemetry included both tympanometries at varying pressures (+200 to −400 dapa). Then, they were elicited ipsilaterally at 1000 and 2000 Hz and contralaterally using pure tones of 500, 1000, 2000, and 4000 Hz.

TEOAEs were induced by clicks (80 dB pe SPL) and analyzed at 1, 1. 5, 2, 3, and 4 kHz in a 20-ms window and were considered present if the response signal to noise ratio (SNR) was 6 dB with reproducibility >70% at three frequencies, with an overall SNR of 6 dB SPL and an overall reproducibility >70%. DPOAEs were in the form of a DP-Gram over f2 750, 984, 1500, 2016, 3000, 3984, 6000, and 7969 Hz (L1 = 65 dB SPL and L2 = 55 dB SPL, f2/f1 = 1. 22). They were considered present if SNR was 6 dB at four frequencies. DPOAE response levels were provided for f2 but were measured at 2f1-f2.

This study was conducted on 30 patients with ISSNHL after excluding six patients (two were diagnosed with vestibular schwannoma, and four were lost to follow-up). The patients' mean age was 42.33 ± 5.54 years and ranged from 20 to 50 years. Eighteen patients (60%) were males, and 12 (40%) were females. All patients were presented with unilateral ISSNHL; 14 (46.7%) in the right ear and 16 (53.3%) in the left ear.

Hearing loss was mild in 14 patients (46.7%), moderate in 4 (13.3%), moderately severe in 4 (13.3%), severe in 6 (20%), and profound in 2 (6.7%). Audiometric configuration was high frequency ISSNHL in 18 patients (60%), flat in 8 (26.7%), and low frequency in 4 (13.3%).

## 4 Statistical analysis

Data were analyzed using SPSS version 23. Means with standard deviations (SD) were used for descriptive statistics. Repeated measures analysis of variance (ANOVA) or Friedman's test compared numerical data for more than two related groups, followed by post-hoc tests with Bonferroni correction. Pearson's and Spearman's correlation coefficients were used for correlation analysis. All P-values less than 0.05 were considered significant.

## 5 Results

TEOAEs were detected in 18 patients during the initial evaluation but were not found in 12. They were detected in 21 patients after one week of treatment and 24 patients after one and three months. DPOAEs were detected in 20 patients at the initial evaluation but were absent in ten. They were detected in 22 patients after one week of treatment and 25 patients after one and three months.

In fourteen mild ISSNHL patients, TEOAEs and DPOAEs were initially detected in 10 patients and were absent in four. In four moderate ISSNHL patients, TEOAEs were initially detected in two patients and were absent in the other two, while DPOAEs were detected in three of four patients. Also, in four moderately severe ISSNHL patients, TEOAEs were initially detected in two patients and were absent in the other two, while DPOAEs were detected in three of four patients. In severe ISNHL patients, TEOAEs and DPOAEs were initially detected in four patients and were absent in two. During the initial assessment, two patients were found to have profound ISSNHL with no TEOAEs or DPOAEs.

Throughout the study, significant improvements in the hearing thresholds at 250–8000 Hz were observed. In contrast, no significant improvements were detected in the word recognition scores. The hearing thresholds at 500–4000 Hz improved significantly at one month and three months compared to the initial evaluation. No statistically significant changes in hearing thresholds and word recognition scores were observed at three months compared to one month (Table 1).

**Table 1:** Pure tone audiometry and word recognition scores at different follow up times.

Items	First evaluation N = 30	After one week N = 30	After one month N = 30	After three months N = 30	Test of sig.
250 Hz Mean ± SD	47. 67 ± 25. 32	38 ± 19. 98	33. 33 ± 20. 40	34 ± 20. 69	F = 2. 780 P = 0. 044∗
500 Hz Mean ± SD	50 ± 25.86	40 ± 20.59	32. 33 ± 20.42 a	32 ± 21.24 a	F = 4. 318 P = 0. 006∗
1000 Hz Mean ± SD	48. 33 ± 26.04	37. 33 ± 20.92	32 ± 22.42 a	32 ± 23.03 a	F = 3. 311 P = 0. 023∗
2000 Hz Mean ± SD	50. 33 ± 25.22	38. 67 ± 20.63	34. 67 ± 20.55 a	33. 67 ± 21.45 a	F = 3. 607 P = 0. 016∗
4000 Hz Mean ± SD	59. 67 ± 29.33	48. 33 ± 21.23	42. 33 ± 22.43 a	41. 67 ± 23.09 a	F = 3. 552 P = 0. 017∗
8000 Hz Mean ± SD	66. 67 ± 31.11	57. 67 ± 27.72	48 ± 29.70	47. 67 ± 30.84	F = 2. 757 P = 0. 046∗
Word recognition% Mean ± SD	72. 27 ± 30.75	84. 27 ± 24.79	87. 47 ± 24.76	86. 40 ± 25.11	F = 2. 207 P = 0. 103

R = Repeated measures ANOVA (Friedman test) ∗ statistically significant.

a: significantly different from the first evaluation value.

b: significantly different from the first-week value.

c: significantly different from the first-month value.

Throughout the study, the transient evoked otoacoustic emissions (TEOAEs) SNR significantly increased at 1, 1.2, 1.5, 2, 3.4, and 4 kHz. In contrast, no significant increase was observed at 3 kHz. Transient evoked otoacoustic emissions (TEOAEs) SNR significantly increased at 1, 1.2, 1.5, 2, and 3.4 kHz at one month and three months compared to the initial evaluation. In addition, it significantly increased at 2 kHz at three months compared to the first week. No significant changes were observed at three months compared to one month (Table 2).

**Table 2:** Transient evoked otoacoustic emissions SNR at different follow-up times.

Items	First evaluation N = 30	After one week N = 30	After one month N = 30	After three months N = 30	Test of sig.
1 kHz Mean ± SD	−0. 14 ± 3. 69	2. 08 ± 3. 51	4. 33 ± 5. 79 a	4. 53 ± 6. 01 a	F = 6. 055 P = 0. 001∗
1. 5 kHz Mean ± SD	1. 90 ± 5. 28	2. 89 ± 3. 62	5. 99 ± 5. 94 a	6. 06 ± 6. 21 a	F = 4. 763 P = 0. 004∗
2 kHz Mean ± SD	1. 19 ± 2. 92	1. 84 ± 2. 94	3. 69 ± 3. 71 a	4. 38 ± 2. 86 a, b	F = 6. 946 P < 0. 001∗
3 kHz Mean ± SD	1. 95 ± 3. 56	3. 30 ± 3. 99	4. 37 ± 4. 67	4. 40 ± 4. 68	F = 2. 224 P = 0. 089
4 kHz Mean ± SD	4. 05 ± 4. 45	4. 02 ± 4. 64	6. 34 ± 4. 42	6. 44 ± 4. 37	F = 2. 773 P = 0. 045∗
1. 2–3. 4 kHz Mean ± SD	2. 28 ± 4. 25	3. 54 ± 3. 79	5. 88 ± 4. 95 a	6. 15 ± 5. 09 a	F = 5. 049 P = 0. 003∗

R = Repeated measures ANOVA (Friedman test) ∗ Significant.

a: Significantly different from the first evaluation value.

b: significantly different from the first-week value.

c: significantly different from the first-month value.

There were negative correlations between hearing thresholds and TEOAEs SNR at 1 kHz at the initial evaluation, one week, one month, and three months. Furthermore, significant negative correlations were found at 2 kHz at the initial evaluation, one month, and three months, with an insignificant negative correlation at one week. In addition, statistically significant negative correlations were detected at 4 kHz at one month and three months, with an insignificant negative correlation at the initial evaluation and one week. Furthermore, negative correlations were noted between the average PTA and TEOAEs SNR at the initial evaluation, one week, one month, and three months (Table 3).

**Table 3:** Correlation between PTA and TEOAEs SNR at different follow-up times.

Items	First Evaluation		After one week		After one month		After three months	
	r	P	R	P	r	P	r	P
PTA vs TEOAEs SNR at 1 kHz	−0. 420	0. 021∗	−0. 399	0. 029∗	−0. 430	0. 018∗	−0. 438	0. 015∗
PTA vs TEOAEs SNR at 2 kHz	−0. 474	0. 008∗	−0. 339	0. 067	−0. 485	0. 007∗	−0. 677	<0.001∗
PTA vs TEOAEs SNR at 4 kHz	−0. 249	0. 184	−0. 335	0. 071	−0. 525	0. 003∗	−0. 598	<0.001∗
Average PTA vs Average TEOAEs SNR	−0. 553	0. 002∗	−0. 456	0. 011∗	−0. 620	<0.001∗	−0. 680	<0.001∗

**r:** Person's correlations coefficient ∗ Significant.

Compared to the initial evaluation, patients with TEOAEs had improved hearing thresholds at 250–8000 Hz and word recognition scores at one month and three months. Also, significant improvements in the hearing thresholds at 2000 Hz and word recognition scores were noted at one week compared to the initial evaluation (Table 4).

**Table 4:** Pure tone audiometry and word recognition scores in patients with present TEOAEs at the first evaluation at different follow-up times.

Items	First evaluation N = 18	After one week N = 18	After one month N = 18	After three months N = 18	Test of sig.
250 Hz Mean ± SD	41. 11 ± 22. 72	31. 67 ± 15. 34	24. 44 ± 9. 84 a	25 ± 11. 63 a	F = 4. 413 P = 0. 007∗
500 Hz Mean ± SD	41. 11 ± 20. 69	31. 11 ± 14. 30	23. 89 ± 8. 32 a	23. 33 ± 10 a	F = 6. 117 P = 0. 001∗
1000 Hz Mean ± SD	41. 11 ± 22. 98	28. 89 ± 11. 83	22. 78 ± 7. 71 a	23. 33 ± 9. 07 a	F = 6. 446 P = 0. 001∗
2000 Hz Mean ± SD	46. 11 ± 19. 22	33.33 ± 13.93 a	25 ± 7. 28 a	24. 44 ± 8. 56 a	F = 10. 678 P < 0. 001∗
4000 Hz Mean ± SD	53. 33 ± 23. 51	41. 11 ± 16. 05	32. 22 ± 14. 78 a	31. 67 ± 15.90 a	F = 5. 772 P = 0. 001∗
8000 Hz Mean ± SD	62. 78 ± 28. 09	50 ± 22. 49	35 ± 20. 44 a	35. 56 ± 23.45 a	F = 5. 596 P = 0. 002∗
Word recognition% Mean ± SD	79. 11 ± 20. 14	92. 44 ± 8.10 a	96. 89 ± 4. 66 a	96 ± 6. 14 a	F = 9. 181 P < 0. 001∗

R = Repeated measures ANOVA (Friedman test) ∗ Significant.

a: Significantly different from the first evaluation value.

b: Significantly different from the first-week value.

c: Significantly different from the first-month value.

Patients with non-detectable TEOAEs had non-significant improvements in hearing thresholds at 250–8000 Hz and word recognition scores throughout the study (Table 5).

**Table 5:** Pure tone audiometry and word recognition scores in patients with absent TEOAEs at the first evaluation at different follow-up times.

Items	First evaluation N = 12	After one week N = 12	After one month N = 12	After three months N = 12	Test of sig.
250 Hz Mean ± SD	57. 50 ± 26. 76	47. 50 ± 22. 91	46. 67 ± 24. 98	45. 83 ± 25. 83	F = 0. 565 P = 0. 641
500 Hz Mean ± SD	63. 33 ± 27. 91	51. 67 ± 23. 29	45. 83 ± 26. 01	45. 83 ± 26. 01	F = 1. 222 P = 0. 313
1000 Hz Mean ± SD	59. 17 ± 27. 54	48. 33 ± 26. 74	45 ± 30. 15	45 ± 30. 15	F = 0. 657 P = 0. 583
2000 Hz Mean ± SD	56. 67 ± 32. 15	48. 33 ± 28. 39	45. 83 ± 27. 87	48. 33 ± 28. 39	F = 0. 314 P = 0. 815
4000 Hz Mean ± SD	69. 17 ± 35. 34	57. 50 ± 25	53. 33 ± 27. 91	53. 33 ± 26. 05	F = 0. 807 P = 0. 497
8000 Hz Mean ± SD	72. 50 ± 35. 64	67. 50 ± 32. 72	63. 33 ± 35. 89	57. 50 ± 31. 73	F = 0. 419 P = 0. 740
Word recognition% Mean ± SD	60. 67 ± 39. 67	71. 33 ± 35. 24	73. 33 ± 35. 42	73. 33 ± 35. 42	F = 0. 333 P = 0. 802

R = Repeated measures ANOVA (Friedman test) ∗ Significant.

a: significance relative to the first evaluation value.

b: significance relative to the first-week value.

c: significance relative to the first-month value.

DPOAEs SNR at 7969, 3984, 3000, 2016, 1500, 984, and 750 Hz demonstrated a significant increase throughout the study, with an insignificant change at 6000 Hz. Also, significant increases were reported at 7969, 3984, 3000, 2016, 1500, 984, and 750 Hz at one month and three months compared to the initial evaluation. In contrast, no significant changes were reported at three months compared to one month (Table 6).

**Table 6:** Distortion product otoacoustic emission SNR at different follow up times.

Items	First evaluation N = 30	After one week N = 30	After one month N = 30	After three months N = 30	Test of sig.
7969 Hz Mean ± SD	0. 33 ± 6. 03	1. 81 ± 5. 68	5. 24 ± 6. 99 a	5. 15 ± 7. 32 a	F = 4. 240 P = 0. 007∗
6000 Hz Mean ± SD	3. 32 ± 5. 76	2. 11 ± 7. 01	4. 83 ± 8. 38	5. 80 ± 7. 67	F = 1. 510 P = 0. 216
3984 Hz Mean ± SD	1. 41 ± 5. 94	3. 14 ± 6. 05	5. 66 ± 5. 10 a	5. 53 ± 5. 39 a	F = 3. 958 P = 0. 010∗
3000 Hz Mean ± SD	2. 01 ± 4. 58	4. 06 ± 3. 47	6. 93 ± 6. 57 a	7. 19 ± 7. 05 a	F = 5. 824 P = 0. 001∗
2016 Hz Mean ± SD	2. 42 ± 8. 75	4. 91 ± 4. 50	8. 75 ± 7. 05 a	9. 05 ± 6. 94 a	F = 6. 279 P = 0. 001∗
1500 Hz Mean ± SD	4. 59 ± 6. 48	5. 65 ± 6. 15	9. 98 ± 7. 21 a	10. 04 ± 6. 99 a	F = 5. 420 P = 0. 002∗
984 Hz Mean ± SD	0. 32 ± 6. 73	3. 65 ± 4. 48	6. 75 ± 5. 66 a	7. 19 ± 5. 21 a	F = 9. 801 P < 0. 001∗
750 Hz Mean ± SD	2. 19 ± 5. 55	4. 17 ± 5. 21	6. 57 ± 5. 86 a	6. 59 ± 6. 01 a	F = 4. 220 P = 0. 007∗

R = Repeated measures ANOVA (Friedman test) ∗ Significant.

a: Significantly different from the first evaluation value.

b: Significantly different from the first-week value.

c: Significantly different from the first-month value.

Significant negative correlations were reported between hearing thresholds at 1000 Hz and DPOAEs SNR at 984 Hz at the initial evaluation, one month, and three months, with an insignificant negative correlation at one week. Also, significant negative correlations were observed between hearing thresholds at 2000 Hz and DPOAEs SNR at 2016 Hz at one month and three months, with insignificant negative correlations at the initial evaluation and one week. At the initial evaluation, significant negative correlations were reported between hearing thresholds at 4000 Hz and DPOAEs SNR at 3984 Hz, one week, one month, and three months. Furthermore, significant negative correlations were noted between hearing thresholds at 8000 Hz and DPOAEs SNR at 7969 Hz at the initial evaluation, one month, and three months, with an insignificant negative correlation at one week (Table 7).

**Table 7:** Correlation between PTA and DPOAEs SNR at different follow-up times.

Items	First Evaluation		After one week		After one month		After three months	
	R	P	R	P	r	P	r	P
PTA at 1000 Hz vs DPOAEs SNR at 984 Hz	−0. 482	0. 007∗	−0. 276	0. 139	−0. 444	0. 014∗	−0. 603	<0.001∗
PTA at 2000 Hz vs DPOAEs SNR at 2016 Hz	−0. 303	0. 103	−0. 241	0. 200	−0. 519	0. 003∗	−0. 479	0. 007∗
PTA at 4000 Hz vs DPOAEs SNR at 3984 Hz	−0. 492	0. 006∗	−0. 467	0. 009∗	−0. 668	<0.001∗	−0. 597	<0.001∗
PTA at 8000 Hz vs DPOAEs SNR at 7969 Hz	−0. 406	0. 026∗	−0. 287	0. 124	−0. 623	<0.001∗	−0. 595	0. 001∗

**r:** Person's correlations coefficient ∗ Significant.

Patients with DPOAEs demonstrated significantly improved hearing thresholds at 250–8000 Hz and word recognition scores. Significant decreases (improvements) in hearing thresholds at 250–8000 Hz and increases (improvements) in word recognition scores were observed at one month and three months compared to the initial evaluation. Also, a significant decrease (improvement) in the hearing threshold at 1000 Hz and an increase (improvement) in speech discrimination scores were noted at one week compared to the initial evaluation. In addition, a significant decrease (improvement) in the hearing threshold at 500 Hz was reported at three months compared to one week (Table 8).

**Table 8:** Pure tone audiometry and word recognition scores in patients with present DPOAEs at the first evaluation at different follow-up times.

Items	First evaluation N = 20	After one week N = 20	After one month N = 20	After three months N = 20	Test of sig.
250 Hz Mean ± SD	36. 50 ± 13.77	29 ± 7. 88	23. 50 ± 6.51 a	23. 50 ± 6. 90 a	F = 8. 869 P < 0. 001∗
500 Hz Mean ± SD	38.50 ± 15.40	31 ± 9. 40	23. 50 ± 6.51 a	22.50 ± 6.59 a, b	F = 10. 807 P < 0. 001∗
1000 Hz Mean ± SD	38 ± 19. 63	28 ± 9. 51 a	21. 50 ± 5.16 a	21. 50 ± 5. 16 a	F = 9. 188 P < 0. 001∗
2000 Hz Mean ± SD	41 ± 17. 14	32.50 ± 15.26	25 ± 6.07 a	25 ± 6. 88 a	F = 7. 605 P < 0. 001∗
4000 Hz Mean ± SD	48.50 ± 20.01	40 ± 15. 89	32.50 ± 11.98 a	31. 50 ± 12. 78 a	F = 5. 187 P = 0. 003∗
8000 Hz Mean ± SD	58 ± 27. 26	48.50 ± 21.03	33. 50 ± 14.52 a	32. 50 ± 14. 55 a	F = 7. 556 P < 0. 001∗
Word recognition% Mean ± SD	84.40 ± 20.31	95.20 ± 6.82a	98. 40 ± 3. 28 a	98 ± 4. 21 a	F = 7. 056 P < 0. 001∗

R = Repeated measures ANOVA (Friedman test) ∗ Significant.

a: Significantly different from the first evaluation value.

b: Significantly different from the first-week value.

c: Significantly different from the first-month value.

Throughout the study, patients with non-detectable DPOAEs had non-significant decreases (improvements) in the hearing thresholds at 250–8000 Hz or increases (improvements) in word recognition scores (Table 9).

**Table 9:** Pure tone audiometry and word recognition scores in patients with absent DPOAEs at the first evaluation at different follow-up times.

Items	First evaluation N = 10	After one week N = 10	After one month N = 10	After three months N = 10	Test of sig.
250 Hz Mean ± SD	70 ± 28. 87	57 ± 23. 94	53 ± 24. 18	53 ± 25. 52	F = 0. 983 P = 0. 412
500 Hz Mean ± SD	73 ± 27. 81	60 ± 25. 60	51 ± 26. 54	52 ± 26. 37	F = 1. 461 P = 0. 241
1000 Hz Mean ± SD	69 ± 25. 69	57 ± 25. 73	54 ± 28. 56	54 ± 29. 51	F = 0. 678 P = 0. 571
2000 Hz Mean ± SD	69 ± 29. 14	56 ± 29. 70	52 ± 30. 29	51 ± 29. 33	F = 0. 783 P = 0. 511
4000 Hz Mean ± SD	82 ± 33. 10	65 ± 27. 28	59 ± 23. 90	58 ± 24. 18	F = 1. 647 P = 0. 196
8000 Hz Mean ± SD	84 ± 32. 39	76 ± 28. 75	71 ± 30. 98	72 ± 31. 02	F = 0. 368 P = 0. 777
Word recognition% Mean ± SD	48 ± 34. 56	58 ± 31. 61	63. 20 ± 33. 98	63. 20 ± 33. 98	F = 0. 456 P = 0. 715

R = Repeated measures ANOVA (Friedman test) ∗ Significant.

a: Significantly different from the first evaluation value.

b: Significantly different from the first-week value.

c: Significantly different from the first-month value.

There was a significant positive correlation between TEOAEs and DPOAEs in almost all correlated frequencies at one week, one, and three months after treatment (Table 10).

**Table 10:** Correlation between TEOAEs and DPOAEs at different follow-up times.

Items	First evaluation		After one week		After one month		After three months	
	r_s_	P	r_s_	P	r_s_	P	r_s_	P
TEOAEs at 1 KHz and DPOAEs at 984 Hz	0.211	0.262	0.766	<0.001∗	0.558	0.001∗	0.737	<0.001∗
TEOAEs and DPOAEs at 1.5 KHz	−0.271	0.148	0.490	0.006∗	0.467	0.009∗	0.467	0.009∗
TEOAEs at 2 KHz and DPOAEs at 2016 Hz	0.373	0.042∗	0.703	<0.001∗	0.470	0.009∗	0.615	<0.001∗
TEOAEs and DPOAEs at 3 KHz	0.336	0.070	0.534	0.002∗	0.510	0.004∗	0.568	0.001∗
TEOAEs at 4 KHz and DPOAEs at 3984 Hz	−0.362	0.050∗	0.324	0.081	0.466	0.010∗	0.513	0.004∗

**r**_**s**_**:** Spearman's correlations coefficient ∗ significance.

## 6 Discussion

The current study included 30 patients with unilateral ISSNHL. No patient had bilateral involvement, which is consistent with **Oh et al. (2007)****,** who stated the rarity of bilateral involvement (less than 2–5%).

The high-frequency SNHL was the most common audiometric configuration observed in the current study, followed by the flat and low-frequency types, and this finding is supported by several studies. **Hussiny et al. (2018)** reported the factors leading to higher vulnerability of the basal region to damage than the apical one and classified them into intrinsic and extrinsic. The intrinsic factors include the differential characteristics of basal outer hair cells, as they have a significantly lower level of the antioxidant glutathione than the apical OHCs. Another intrinsic factor is the difference in Ca^++^ homeostasis in the OHCs of the cochlear base and apex. Three factors determine OHCs cytoplasmic Ca^++^ homeostasis; the first is Ca^++^ influx into OHCs via mechanotransducer (MET) channels; the second is the buffering of Ca^++^ cytoplasmic load by calcium-binding proteins and organelles like mitochondria; the third is Ca^++^ extrusion by the plasma membrane Ca ATPase pump. High-frequency basal OHCs are more susceptible to damage and apoptosis than apical OHCs due to their preferential increased risk of intracellular Ca^++^ overload. ue to the small membrane area, the basal ones have higher MET currents and lower Ca++ extrusion rates.

The extrinsic factors are based on the hypothesis of different etiologies as acoustic trauma hits the basal turn of the cochlea with more energy load than the relatively protected apical region by the acoustic reflex. Furthermore, when viruses infect the middle ear or round window niche, they first reach the anatomically closed area of the basal part (**Hussiny et al., 2018****).**

The current study has revealed statistically significant improvements in hearing thresholds at 250–8000 Hz. This finding is in line with **Hara et al. (2018)****,** who reported improvement in PTA by 13.8 ± 16.6 dB for 31 patients treated within two weeks of ISSNHL onset by corticosteroids. Also, **Slattery et al. (2005)** detected a statistically significant improvement in the four-frequency pure-tone average and speech discrimination score at one month following four intratympanic injections of methylprednisolone.

This study reported significant increases in TEOAEs SNR at 1, 1.2, 1.5, 3.4, and 4 kHz. In line with this finding**,****Nemati et al. (2011)** found a significant and positive change in the overall TEOAE SNR and reproducibility following treatment for patients with considerable hearing recovery. TEOAEs and DPOAEs reflect the OHCs activity. OHC function deteriorates when the hearing threshold is raised and recovers as hearing improves **(****Nemati et al., 2011****).**

Hearing thresholds and TEOAEs SNR at 1 kHz were found to have statistically significant negative correlations at the initial evaluation, one week, one month, and three months. In addition, statistically significant negative correlations were reported at 2 kHz at the initial evaluation, one month, and three months. In addition, statistically significant negative correlations were detected at 4 kHz at one month and three months. Furthermore, statistically significant negative correlations were reported between the average PTA and TEOAEs SNR at the initial evaluation, one week, one month, and three months. These findings are consistent with **Nakamura et al. (1997)****,** who revealed a concurrent increase in TEOAEs and DPOAEs amplitudes with the hearing threshold recovery in 15 patients with ISSNHL. These findings suggest outer hair cell function deterioration when the hearing threshold is elevated and recovery as hearing improves.

In contrast, **Truy et al. (1993)** illustrated that correlations between hearing recovery and TEOAE amplitude were insufficient to guarantee the clinical use of TEOAE in predicting outcomes in ISSNHL patients.

In the current study, significant improvements were observed in hearing thresholds and word recognition scores in patients with TEOAEs. In contrast, no significant improvements were detected in patients with no TEOAEs. **Lalaki et al. (2001)** agreed with these results. They detected TEOAEs or acceptable peak amplitudes in at least some frequency bands during the first two measurements in 61% of patients with recovered hearing. Additionally, **Shupak et al. (2014)** found that many patients with measurable TEOAEs at the second follow-up had more than 50% hearing improvement at the three-month follow-up.

Nevertheless, according to **Hoth (2005)****,** small and large amplitude TEOAEs were measurable in normal and hearing-impaired ears, respectively. TEOAEs were more robust in some cases than expected at corresponding hearing thresholds, particularly at 1000, 1500, and 2000 Hz. Similarly, **Canale et al. (2005)** observed hearing recovery in most patients with present TEOAEs. However, the results were insignificant to suggest that TEOAEs could be used to predict prognosis in low-frequency SNHL patients.

In this study, DPOAE SNR significantly increased at 7969, 3984, 3000, 2016, 1500, 984, and 750 Hz. However, a non-significant increase was reported at 6000 Hz, which may be due to a more significant cochlear cell impairment at the mid frequencies than the higher ones.

Significant negative correlations were reported between hearing thresholds at 1000 Hz and DPOAEs SNR at 984 Hz at the initial evaluation, one month, and three months. Also, hearing thresholds at 2000 Hz were negatively correlated with DPOAEs SNR at 2016 Hz at one month and three months. In addition, significant negative correlations were observed between hearing thresholds at 4000 Hz and DPOAEs SNR at 3984 Hz at the initial evaluation, one week, one month, and three months. Furthermore, hearing thresholds at 8000 Hz were negatively correlated with DPOAEs SNR at 7969 Hz at the initial evaluation, one month, and three months.

The results of **Bashiruddin et al. (2018)** are consistent with these results, as they revealed improvement in the hearing thresholds at all measured frequencies (1500–12000 Hz). DPOAE SNR significantly changed at 1500, 2000, and 8000 Hz. Moreover, SNR change and hearing threshold change showed significant associations at 8000 and 10000 Hz.

Significant improvements in hearing thresholds and word recognition scores were noted in patients with DPOAEs, whereas no significant improvements were detected in patients with no DPOAEs. In line with these findings, **Shupak et al. (2014)** revealed that patients with TEOAEs had an average hearing improvement of 62 ± 41% on the first follow-up, while those with no response improved by 11 ± 15%. Furthermore, a significant DPOAE shortly after acute damage is associated with a better hearing prognosis, according to **Park et al. (2010)**, even with considerably elevated hearing thresholds. **Schweinfurth et al. (1997)** used steroids to treat three patients with DPOAEs. Their PTA at 500–2000 Hz improved by 33 dB. In contrast, five of seven individuals with no DPOAEs had no improvement despite steroid medication. **Liu et al. (2022)** stated that DPOAE detection within three days of treatment indicates a favorable outcome or, at the very least, the possibility of a cure.

The findings of **Nemati et al. (2011)** are inconsistent with the current results. They concluded that patients with substantial hearing recovery did not significantly change in DPOAE SNR or amplitude after treatment.

In the current study, the positive correlations between the TEOAEs and DPOAEs in ISSNHL patients at one week, one, and three months of treatment may be attributed to OHC and cochlear function recovery.

OAEs are objective rapid tests with good reliability and stability (Keppler et al., 2010; Kochanek et al., 2015). In the current study, TEOAEs and DPOAEs were absent in 4 of 14 mild ISSNHL patients at the initial evaluation. In contrast, they were detected in moderately severe (2 of 4 cases) and severe patients (4 of 6 cases), who had better outcomes than mild cases with no emissions. The presence of OAEs indicates a good prognosis irrespective of the hearing loss degree. Also, OAEs presence during treatment and follow-up has a significant prognostic value.

There were significant improvements in hearing thresholds throughout the study at 250–8000 Hz. After therapy, hearing improvement was more pronounced in patients with TEOAEs and DPOAEs than in others with no emissions. As a result, TEOAEs and DPOAEs are recommended as routine tests in all SSNHL patients to predict outcomes and monitor treatment as TEOAEs and DPOAEs reflect the cochlear OHCs activity.

## Author statement

**1- Aya El-sayed El-sayed Gaafar,** Master.

Clinical data collection, analysis and writing of the paper.

**2-Elshahat Ibrahem Ismail**, MD.

Clinical data collection, analysis and writing of the paper.

**3- Hesham Saad Zaghloul**, MD.

Clinical data collection, analysis and writing of the paper.

## Declaration of competing interest

All the authors declare that they have not any conflict of interest.

## References

[bib1] Babich K., Dunckley K.T. (2020). Idiopathic sudden sensorineural hearing loss: should otoacoustic emissions Be added to the monitoring protocol? A systematic review. Appl. Sci..

[bib2] Bashiruddin J., Risdawati Bramantyo B., Bardosono S. (2018). Proceedings of the 2nd Physics and Technologies in Medicine and Dentistry Symposium.

[bib3] Canale A., Lacilla M., Giordano C., De Sanctis A., Albera R. (2005). The prognostic value of the otoacoustic emission test in low frequency sudden hearing loss. Eur. Arch. Oto-Rhino-Laryngol..

[bib4] Chau J.K., Lin J.R.J., Atashband S., Irvine R.A., Westerberg B.D. (2010). Systematic review of the evidence for the aetiology of adult sudden sensorineural hearing loss. Laryngoscope.

[bib5] Chen C.N. (2006). Differentiating the cause of acute sensorineural hearing loss between Meniere's disease and sudden deafness. Acta Otolaryngol..

[bib6] Choung Y.H., Park K., Shin Y.R., Cho M.J. (2009). Intratympanic dexamethasone injection for refractory sudden sensory neural hearing loss. Laryngoscope.

[bib7] Gunes A., Karali E., Ural A., Ruzgar F., Bayatkara T. (2019). The relationship of high-frequency distortion product otoacoustic emission (DPOAE) values with hematological parameters in tinnitus patients. Eur Arch Otorhinolaryngol..

[bib8] Hara J.H., Zhang J.A., Gandhi K.R., Flaherty A., Barber W., Leung M.A., Burgess L.P. (2018). Oral and intratympanic steroid therapy for idiopathic sudden sensorineural hearing loss. Laryngoscope Invest. Otolaryngol..

[bib9] Hoth S. (2005). On a possible prognostic value of otoacoustic emissions: a study on patients with sudden hearing loss. Eur. Arch. Oto-Rhino-Laryngol..

[bib10] Hussiny A.M., Abou-Halawa A.S., Elnahriry T.A., Abou-Setta A.E. (2018).

[bib11] Huy P., Sauvaget E. (2005). Idiopathic sudden sensorineural hearing loss is not an otologic emergency. Otol. Neurotol..

[bib12] Ishida I., Sugiura M., Teranishi M., Katayama N., Nakashima T. (2008). Otoacoustic emissions, ear fullness and tinnitus in the recovery course of sudden deafness. Auris Nasus Larynx.

[bib13] Jedrzejczak W.W., Pilka E., Ganc M., Kochanek K., Skarzynski H. (2022). Ultra-high frequency distortion product otoacoustic emissions for detection of hearing loss and tinnitus. Int. J. Environ. Res. Publ. Health.

[bib14] Keppler H., Dhooge I., Maes L., D'haenens W., Bockstael A., Philips B., Swinnen F., Vinck B. (2010). Transient-evoked and distortion product otoacoustic emissions: a short-term test-retest reliability study. Int. J. Audiol..

[bib15] Kochanek K.M., Śliwa L.K., Puchacz K., Piłka A. (2015). Repeatability of transient-evoked otoacoustic emissions in young adults. Med. Sci. Mon. Int. Med. J. Exp. Clin. Res..

[bib16] Kuhn M., Heman-Ackah S.E., Shaikh J.A., Roehm P.C. (2011). Sudden sensorineural hearing loss: a review of diagnosis, treatment, and prognosis. Trends Amplif..

[bib17] Lalaki P., Markou K., Tsalighopoulos M., Danilidis I. (2001). Transiently evoked otoacoustic emissions as a prognostic indicator in idiopathic sudden hearing loss. Scand. Audiol..

[bib18] Lonsbury-Martin B.L., Martin G.K. (2003). Otoacoustic emissions. Curr. Opin. Otolaryngol. Head Neck Surg..

[bib19] Liu S.Y., Shi W.Y., Zheng H.Y., Yuan Y.X., Li J.J., Li Q. (2022). The correlation between detection value of distortion-product otoacoustic emissions and the early prognosis of sudden sensorineural hearing loss. J. Int. Adv. Otol..

[bib20] Nakamura M., Yamasoba T., Kaga K. (1997). Changes in otoacoustic emissions in patients with idiopathic sudden deafness. Audiology.

[bib21] Nemati S., Naghavi S., Kazemnejad E., Banan R. (2011). Otoacoustic emissions in sudden sensorineural hearing loss: changes of measures with treatment. Iran. J. Otorhinolaryngol..

[bib22] Nosrati-Zarenoe R., Arlinger S., Hultcrantz E. (2007). Idiopathic sudden sensorineural hearing loss: results drawn from the Swedish national database. Acta Otolaryngol..

[bib23] Oh J.H., Park K., Lee S.J., Shin Y.R., Choung Y.H. (2007).

[bib24] Park H., Lee Y., Park M., Kim J., Na B., Shin J. (2010). Short-term changes of hearing and distortion-product otoacoustic emissions in sudden sensorineural hearing loss. Otol. Neurotol..

[bib25] Schweinfurth J., Cacace T., Parnes S. (1997). Clinical application of otoacoustic emissions in sudden hearing loss. Laryngoscope.

[bib26] Shupak A., Zeidan R., Shemesh R. (2014). Otoacoustic emissions in the prediction of sudden sensorineural hearing loss outcome. Otol. Neurotol..

[bib27] Slattery W., Fisher L., Iqbal Z., Liu N. (2005). Oral steroid regimens for idiopathic sudden sensorineural hearing loss. Otolaryngol. Head Neck Surg..

[bib28] Soliman S. (1976). Speech discrimination audiometry using Arabic phonetically balanced words. Ain Shams Med. J..

[bib29] Soliman S.M., Fathalla A., Shehata M. (1985).

[bib30] Truy E., Veuillet E., Collet L., Morgon A. (1993). Characteristics of transient otoacoustic emissios in patients with sudden idiopathic hearing loss. Br. J. Audiol..

[bib31] Vaden K.I., Matthews L.J., Dubno J.R. (2018). Transient-evoked otoacoustic emissions reflect audiometric patterns of age-related hearing loss. Trends Hear.

[bib32] Vijayendra H., Buggaveeti G., Parikh B., Sangitha R. (2012). Sudden sensorineural hearing loss: an otologic emergency. Indian J. Otolaryngol. Head Neck Surg..

